# P2Y_12_ Receptor Inhibitors in Acute Coronary Syndromes: What Is New on the Horizon?

**DOI:** 10.1155/2013/195456

**Published:** 2013-02-19

**Authors:** Adriana Dana Oprea, Wanda M. Popescu

**Affiliations:** Department of Anesthesiology, Yale University, New Haven, CT 06520, USA

## Abstract

Dual antiplatelet therapy with aspirin and a P2Y_12_ receptor inhibitor represents the cornerstone therapy for patients with acute coronary syndromes or undergoing percutaneous interventions, leading to a reduction of subsequent ischemic events. Variable response to clopidogrel has received close attention, and pharmacokinetic, pharmacodynamic, and pharmacogenomic factors have been identified as culprits. This led to the introduction of newer, potentially safer, and more effective antiplatelet agents (prasugrel and ticagrelor). Additionally, several point-of-care assays of platelet function have been developed in recent years to rapidly screen individuals on antiplatelet therapy. While the routine use of platelet function testing is uncertain and not currently recommended, it may be useful in instances when the degree of platelet inhibition may be uncertain such as high-risk patients undergoing percutaneous coronary intervention or when there may be a suspected pharmacodynamic interaction with other drugs. The current paper focuses on the P2Y_12_ receptor inhibitors and their pharmacogenetics and indications in patients with acute coronary syndromes or receiving percutaneous coronary interventions as well as the applicability of platelet function testing in this clinical context.

## 1. Introduction

The clinical presentation of patients with coronary atherosclerosis is either as stable angina or as an acute coronary syndrome (ACS). The ACSs represent the more acute clinical manifestations of coronary artery disease (CAD) and include unstable angina (UA), non-ST elevation myocardial infarction (NSTEMI), and ST elevation myocardial infarction (STEMI). Despite maximal therapy, 5%–10% of patients with ACS will suffer a recurrent cardiac event or death within the first month after the initial presentation. While patients with stable angina have only narrowing of their coronary arteries, those with ACS have atheromatous plaque rupture and acute thrombus formation. Therefore, it is well recognized that platelet activation and aggregation play a central role in the physiopathology of ACS. In addition to aspirin and anticoagulants, antiplatelet agents represent an important therapeutic step for this patient population, with newer and more potent agents becoming available on the market [1, 2].

During an acute coronary event, vascular wall injury exposes collagen that leads to adhesion of inactive platelets, which subsequently become activated. Platelet activation results in degranulation and secretion of adenosine diphosphate (ADP), thromboxane A2 (T_X_A2), and platelet-activating factor (PAF) [3, 4]. Two G protein-coupled receptors, P2Y_1_ and P2Y_12_, are responsible for platelet aggregation, with the P2Y_1_ receptor initiating a weak platelet activation while binding of P2Y_12_ receptor resulting in a slower but progressive platelet aggregation. 

Several antiplatelet agents are used during an ACS episode, each blocking a different pathway of the platelet aggregation. Aspirin irreversibly inhibits cyclooxygenase COX2, the enzyme that mediates the first step in the biosynthesis of TxA2 from arachidonic acid. The ADP receptor blockers (P2Y_12_ inhibitors) prevent binding of the ADP to a specific platelet receptor, therefore, inhibiting the activation of the GP IIb/IIIa complex and thus platelet aggregation. GP IIb/IIIa antagonists interfere with the final common pathway of platelet aggregation (the cross-bridging of platelets by fibrinogen binding to the GP IIb/IIIa receptor) and may also prevent adhesion of other platelets to the vessel wall. 

This paper focuses on the newer generation P2Y_12_ inhibitors, with the exception of the first generation thienopyridine (ticlopidine), their advantages, limitations, and clinical applicability in the setting of ACS.

## 2. Pharmacokinetics and Pharmacodynamics

### 2.1. Antiplatelet Drugs Available on the Market

#### 2.1.1. Clopidogrel

Clopidogrel is a second-generation thienopyridine introduced after ticlopidine, which irreversibly inhibits the P2Y_12_ receptor and has a bioavailability of 50% after oral absorption. Clopidogrel is a prodrug with no antiplatelet activity of its own. Fifteen percent of the absorbed drug is metabolized by the liver in a 2-step process into an active metabolite (R130964) responsible for the inhibition of platelet aggregation (IPA). The remainder 85% is transformed by esterases into an inactive carboxylic acid metabolite (SR 26334). 

Doses up to 600 mg lead to a peak plasma level within 1 to 2 hours, but maximal platelet aggregation inhibition can be reached in some cases 4–6 h after a loading dose [5]. The half-life of clopidogrel is 6 hours after a single dose and 8 hours for its active metabolite [6]. Administration of a 75 mg oral dose of clopidogrel results in 40% to 60% level of IPA after 3 to 7 days. A loading dose of 600 mg similar response can lead to similar levels of IPA after 2 hours only [7, 8]. Clopidogrel is equally eliminated in feces and urine. Moderate renal and hepatic impairments do not require dose adjustments [9, 10]. The irreversible binding of the P2Y_12_ receptor results in the antiaggregant effects of clopidogrel lasting for the lifespan of platelet, 7–10 days.

#### 2.1.2. Prasugrel

Prasugrel is a newer generation thienopyridine approved for patients with unstable angina or myocardial infarction who undergo percutaneous coronary intervention (PCI) [11].

Similar to clopidogrel, prasugrel is an irreversible P2Y_12_ inhibitor requiring metabolic activation by intestine and blood esterases. Unlike clopidogrel, it requires only one metabolic step, versus two steps. The resulting metabolite is then oxidized by cytochrome P450 (CYP) to an active metabolite (R-138727) responsible for the antiplatelet effects [12]. Peak plasma concentration is reached within 30 minutes of absorption and elimination half-life is approximately 7 hours [13]. As with clopidogrel, moderate hepatic or renal impairment does not require dose adjustments [14, 15]. The maximum IPA (75% to 85%) is achieved between 2 and 4 hours after a loading dose. Compared to clopidogrel, prasugrel is more potent and has a more rapid onset of action at both loading and maintenance doses [11].

 The recommended loading dose for prasugrel is 60 mg, followed by a 10 mg daily dose. Patients weighing less than 60 kg, older than 75 years, or with a previous history of stroke or transitory ischemic attacks require reduction of the dose to 5 mg daily due to increased risk of fatal bleeding [16, 17].

#### 2.1.3. Ticagrelor

Ticagrelor (AZD6140) is a selective, reversible P2Y_12_ inhibitor that belongs to a new class of antiplatelet agents, the cyclopentyl-triazolo-pyrimidines. It is approved for use in combination with low-dose aspirin to reduce the rate of thrombotic cardiovascular events in patients with acute coronary syndrome (ACS). In addition to antiplatelet effects, ticagrelor blocks the ADP-mediated vasoconstriction and is thought to increase the adenosine-induced coronary blood flow [18–20].

In addition to being a reversible antiplatelet agent, ticagrelor is also a noncompetitive antagonist of the P2Y_12_ receptor. Ticagrelor does not directly inhibit ADP binding to the P2Y_12_ receptor but induces a conformational change making it unable to trigger platelet activation [20]. Since it does not require any biotransformation, it is immediately active after oral administration resulting in a more rapid onset of action and a more pronounced platelet inhibition as compared to other P2Y_12_ inhibitors [21]. Ticagrelor's effects correlate with drug plasma levels, its activity being independent of the ADP concentration [22].

Both ticagrelor and its metabolite have linear pharmacokinetics up to a dose of 100 mg twice a day [20]. Ticagrelor is excreted in the feces and it does not require dose adjustments in patients with renal failure [23]. Unlike clopidogrel or prasugrel, patients with impaired liver function or who take medications able to inhibit the CYP3A4 should receive a reduced dose of ticagrelor [22]. Ticagrelor is faster acting and exhibits greater degree of IPA (approximately 80% at 2 hours) when compared with clopidogrel. Despite its reversibility and rapid onset, it has a slow offset, with 20% inhibition present 3 days after stopping therapy. Compared to clopidogrel, there is a faster recovery of the platelet function, with similar IPAs present 5 days after stopping clopidogrel and 3 days after stopping ticagrelor [24–26]. 

Compared to prasugrel, patients with high on-treatment platelet reactivity while on clopidogrel presenting with ACS demonstrated higher IPAs while on ticagrelor [27].

Ticagrelor has a half-life of 7 to 8.5 hours. The recommended loading dose is of 180 mg followed by 90 mg dose twice daily in addition to aspirin 75–100 mg daily [20, 22].

### 2.2. Antiplatelet Drugs in Development

#### 2.2.1. Cangrelor

Cangrelor is a direct-acting, reversible P2Y_12_ antagonist undergoing phase III trials. Unlike the other P2Y_12_ antagonists, cangrelor is administered intravenously. The major therapeutic advantage of this drug is its very short half-life leading to prompt termination of its effect upon stopping the infusion. Initiation of a cangrelor infusion results in a rapid onset of action and an almost complete IPA within 30 minutes. Cangrelor clearance does not depend on renal function. At infusion rates between 2 and 4 *μ*g/kg/min, patients demonstrate an 80% IPA, with platelet function recovering within 5 minutes after infusion discontinuation [28].

#### 2.2.2. Elinogrel

Elinogrel (PRT060128), a quinazoline-2,4-dione is another direct, potent and reversible inhibitor of the platelet P2Y_12_ receptor. Phase II trials have been completed, but further investigations are currently halted. Unlike cangrelor, it is designed to be administered both orally and intravenously. Elinogrel has a plasma half-life of approximately 12 h and achieves almost complete platelet inhibition within 15 minutes after intravenous administration. It does not require metabolization to an active compound and it is excreted equally by the liver and the kidney.

The lack of metabolization to an active substance makes elinogrel less susceptible to resistance, as compared to clopidogrel [29, 30].

## 3. Resistance and Pharmacogenetics

Resistance to antiplatelet agents is a well-described phenomenon, consisting either of presence of inadequate platelet inhibition or the occurrence of a recurrent event while on a therapeutic dose of an antiplatelet agent. More recently, the term resistance has been restricted to the laboratory evidence of high on-treatment platelet reactivity (HTPR), while the clinical presence of a recurrent event has been termed treatment failure [31, 32]. 

Resistance to clopidogrel is a well-recognized phenomenon, with 15–30% of patients having inadequate platelet inhibition while on a therapeutic dose. This poor responsiveness to clopidogrel in certain patient population, as assessed by platelet function testing, has led to studies looking at the potential causes for resistance, the correlation of hypo- or nonresponsiveness to therapy with clinical outcomes (major adverse cardiac events) and at ways to overcome this problem (either by increased dosing or by a prescribing a different antiplatelet agent). 

Several mechanisms are involved in resistance to clopidogrel: inadequate dosing or absorption, drug interactions, and genetic polymorphisms.

Inadequate dosing can be a factor of resistance in obese patients. Absorption can be impaired in patients with cardiogenic shock or with loss of the P-glycoprotein transporter function [33, 34]. Other patient population demonstrating an inadequate response to clopidogrel are the diabetic or insulins resistant patients [35]. In these patients, the normal effects of insulin on the platelet leading to aggregation are impaired. 

Drug interactions represent a major component of clopidogrel resistance. Statins, proton pump inhibitors (PPIs), ketoconazole, and erythromycin interact with different CYP enzymes, thereby decreasing the conversion of clopidogrel to the active metabolite [36–40]. 

Clopidogrel is metabolized to its active form by the CYP enzymes, a super family of microsomal drug-metabolizing enzymes. Functional CYP polymorphisms consist of deletions, duplications, and mutations creating inactive gene products leading to increased or decreased metabolism of the drug. Clopidogrel's metabolism involves 2 steps. The first step leads to the formation of 2-oxo-clopidogrel, which subsequently is transformed into the active metabolite [41]. Enzymes involved in the metabolism of clopidogrel include CYP1A2, CYP2B6, CYP2C9, CYP2C19, and CYP3A4/5. CYP2C19, with its gene located on chromosome 10, seems to be the most important enzyme involved in clopidogrel's metabolism. The CYP2C19∗1 allele is associated with full enzyme activity while CYP2C19∗2 and ∗3 variants are most frequently associated with poor responsiveness to clopidogrel. These alleles have been termed “loss of function alleles” and are more frequent in the Asian rather than in the Caucasians population. Many studies have confirmed an impaired response to clopidogrel either in healthy volunteers or patients carrying the CYP2C19∗2 or ∗3 allele, evidenced by platelet function tests or by an increased incidence of MACE [41, 42] (Table 1). More importantly, the difference in clopidogrel metabolism appears to be present not only in homozygous patients but also heterozygote patients [43, 44]. Due to this evidence, the FDA has added a black box warning for patients with genetic variants of the CYP2C19 gene who are poor metabolizers and receive clopidogrel therapy. These patients are at particular high risk of a poor response to therapy and thus an increased rate of cardiovascular events [45].

On the other hand, the ∗17 allele (gain-of-function allele) is associated with CYP enzyme upregulation, increasing the clopidogrel metabolism by 30%, therefore, leading not only to an adequate antiplatelet response but also to potentially increased bleeding episodes [46–48]. However, a recent meta-analysis did not find any association between the genotype (∗17) and the rate of cardiovascular events or bleeding [49].

ABCB1 is another gene receiving attention as potentially responsible for clopidogrel resistance. ABCB1 encodes the P-glycoprotein efflux transporter responsible for the intestinal absorption of clopidogrel. A certain genetic variant (ABCB1 3435C-T) has been linked to an increased ischemic event while on clopidogrel, but other studies have not confirmed it [50, 51].

Paraoxonase 1 (PON1) is a HDL-associated enzyme that is involved in the second step of clopidogrel metabolism. Its common polymorphism, Q192R, influences platelet inhibition [52]. Recent studies offer contradictory information regarding the association between PON1 polymorphism and a lower degree of platelet aggregation or an increased risk of stent thrombosis [53, 54].

The relationship between polymorphisms of the gene encoding the P2Y_12_ receptor and a response variability to clopidogrel therapy was also studied, but the data remains inconclusive [42].

Resistance to prasugrel is less prevalent, with fewer than 6% of patients qualifying as poor responders on a maintenance dose of prasugrel of 10 mg daily. In the setting of PCI, a significant number of patients did not achieve adequate platelet inhibition after a loading dose of 60 mg of prasugrel, thus, being at an increased risk for MACE [55]. However, as compared to clopidogrel, prasugrel's metabolism depends mostly on CYP3A4 and CYP2B6 enzymes and to a lesser extent on CYP2C9 and CYP2C19 [56–59]. On the other hand, prasugrel's metabolism involves just one step versus 2 steps for clopidogrel. These differences in metabolism as well as its increased potency relative to clopidogrel make prasugrel less susceptible to resistance. Patients demonstrating “resistance” to clopidogrel, based on HTPR, seem to have an adequate response to prasugrel therapy [60–63]. Possible mechanisms responsible for prasugrel resistance include poor patient compliance, drug absorption disturbances, drug interactions (the concomitant use of CYP3A4 inhibitors), drug underdosing, increased platelet turnover, and P2Y_12_ receptor polymorphism [64–66]. 

Ticagrelor is primarily metabolized via the cytochrome CYP3A4 enzyme, which leads to faster, greater, and more consistent antiplatelet effects as compared to clopidogrel. Ticagrelor therapy can also overcome nonresponsiveness in patients with treatment failure while on clopidogrel. Its pharmacodynamics is not influenced by CYP2C19 and ABCB1 genotypes. As such, patients resistant to both clopidogrel and prasugrel seem to be effectively treated with ticagrelor [67, 68].

## 4. Platelet Testing

Platelet function testing, as means to monitor antiplatelet therapy results, has received a lot of attention in the last decade. As detailed above, certain patient populations can be less responsive or even nonresponsive to P2Y_12_ inhibitors (mainly to clopidogrel). In such patients, platelet function assays have been extensively investigated. The main goal was to tailor drug dosing to therapeutic response and therefore prevent MACE. Several platelet function tests are available on the market, each with their own advantages and disadvantages [69]. 

Light transmission aggregometry (LTA) is considered the gold-standard platelet function test. Initial studies showed a good correlation between the residual platelet activity while on clopidogrel and MACE. LTA measures the response of platelets to ADP in platelet-rich plasma. However, it is a method that is labor intensive and operator dependent, and results may not be consistent between laboratories. These limitations led to development of newer tests [32, 70–72] (Table 2).

The VerifyNow P2Y_12_ assay (Accumetrics, San Diego, CA, USA) is a fast, standardized point-of-care platelet test, which measures the platelet-induced aggregation by ADP as an increase in light transmittance in whole blood. The assay contains ADP that induces platelet activation and PGE1 that improves the specificity of detecting P2Y_12_ receptor inhibition. Light transmittance increases due to the fact that activated platelets bind fibrinogen-coated beads. The instrument measures this change in the optical signal and reports results in P2Y_12_ Reaction Units (PRUs). A higher PRU reflects greater ADP-mediated platelet reactivity [70]. Studies have shown increased ischemic event rates and suboptimal platelet inhibition with VerifyNow results reported as PRU > 240 [32, 73, 74].

The vasodilator-stimulated phosphoprotein (VASP) phosphorylation assay represents a highly specific platelet function test. This test measures the inhibition of VASP phosphorylation by ADP, mediated by the P2Y_12_ receptor. Citrated blood is incubated with either prostaglandin E1 (PGE1) or PGE1 and ADP, followed by immunolabeling with CD61 platelet specific antibody and a VASP-P monoclonal antibody or a negative isotopic control antibody.

The platelet reactivity index (PRI) recorded is determined according to a standardized flow cytometric assay [70, 71]. PRI > 50% by VASP analysis is currently the accepted evidence of HTPR [75].

Multiple electrode platelet aggregometry (MEA) is based on impedance aggregometry and measures platelet function in diluted whole blood. Compared to LTA, centrifugation is not necessary and the results can be obtained in approximately 10 minutes. The adhesion of activated platelets to the electrodes leads to an increase of impedance, which is detected for each sensor unit separately and transformed to aggregation units (AU) [70, 72]. The cutoff for HTPR using MEA is 47 AU [76]. 

The INNOVANCE PFA P2Y from the PFA-100 system (Siemens, Marburg, Germany) is a rapid and easy test which uses PGE1 as the AC activator. The presence of the platelet activator and the high shear rates, under standardized conditions, lead to platelet adhesion, activation, and aggregation resulting in the formation of a platelet plug. The device measures the time necessary for a platelet plug to be formed expressed as closure time. A closure time less than 106 s was proposed a measure of clopidogrel resistance in one study [77].

Impact-R (DiaMed, Cressier, Switzerland) measures platelet adhesion and aggregation under high shear conditions [75].

Plateletworks (Helena Laboratories, Beaumont, TX, USA) is another point-of-care monitor of platelet count and function using whole blood. The test is based on platelet aggregation in the presence of a platelet agonist. In patients without platelet dysfunction, the presence of ADP reduces the platelet count to approximately zero, due to the aggregation of most platelets. In contrast, in the presence of platelet inhibition by P2Y_12_ antagonists, not all the platelets aggregate reducing the difference between the initial platelet count and the postagonist count. The ratio between the aggregated platelets in the agonist sample and the platelet count in the reference tube x 100% is used as the degree of platelet aggregation [75, 78]. 

Several studies correlated the results of platelet function tests with the rate of MACE, the most comprehensive one being the POPULAR study (Do Platelet Function Assays Predict Clinical Outcome in Clopidogrel-Pretreated Patients Undergoing Elective PCI). This was a prospective, observational single-center study of 1069 patients undergoing coronary stenting that were treated with clopidogrel. In this trial, the residual platelet function measured by LTA, VerifyNow, VASP, Plateletworks, INNOVANCE PFA P2Y, IMPACT-R assay, and PFA-100 was used to predict MACE and ischemic stroke [79]. Only LTA, VerifyNow, and Plateletworks were significantly associated with the primary end-point. In a prospective study of 1608 patients with CAD, a low response to clopidogrel as assessed with MEA was significantly associated with an increased rate of drug eluting stent thrombosis [80]. Other studies assessing the accuracy of various platelet function tests in predicting MACE in patients on antiplatelet therapy are summarized in Table 3.

A recent prospective study suggests that a therapeutic window for platelet reactivity may be identified using the VerifyNow assay. This “therapeutic window” refers to an “ideal” level of platelet inhibition in which patients would be at a lower risk for both bleeding and ischemic events [81].

## 5. Clinical Applications of the P2Y_12_ Inhibitor Therapy in ACS

Dual antiplatelet therapy with aspirin and a P2Y_12_ receptor inhibitor is a standard therapy for patients with ACS. The clinical advantages and disadvantages of prasugrel and ticagrelor over clopidogrel, as discussed below, led to updates in the ACCF/AHA recommendations [82, 83]. 

Clopidogrel is the most commonly used P2Y_12_ inhibitor in the treatment of ACS. The CURRENT-OASIS 7 trial compared a standard loading dose of 300 mg clopidogrel versus a double loading dose (600 mg) in patients with ACS. When analyzing the entire cohort of patients, the primary outcomes (cardiovascular death, myocardial infarction, and stroke at 30 days), as well as the expanded secondary composite outcome that included recurrent ischemia, did not differ significantly between the two groups. However, in the subset of patients undergoing PCI, the double loading dose clopidogrel resulted in a significantly reduced rate of stent thrombosis [84].

The TRITON-TIMI 38 trial (Trial to Assess Improvement in Therapeutic Outcomes by Optimizing Platelet Inhibition with Prasugrel-Thrombolysis in Myocardial Infarction) compared prasugrel (a 60 mg loading dose and a 10 mg daily maintenance dose) to clopidogrel (a 300 mg loading dose and a 75 mg daily maintenance dose) in patients with ACS who were referred for PCI. Prasugrel therapy was associated with significantly reduced rates of ischemic events (9.9% versus 12.1%, *P* < 0.001), including stent thrombosis, but with an increased risk of major bleeding (2.4% versus 1.8%), including fatal bleeding [16]. 

In a new randomized, double-blinded study of patients with UA or NSTEMI not undergoing revascularization, prasugrel did not significantly reduce the frequency of death from cardiovascular causes, myocardial infarction, or stroke, as compared with clopidogrel, and similar risks of bleeding were observed [85].

A subanalysis of the TRITON-TIMI 38, including only the patients with STEMI, concluded that prasugrel was more effective than clopidogrel for prevention of ischemic events, without an apparent excess in bleeding [86].

Ticagrelor was directly compared to clopidogrel in the PLATO (platlet inhibition and patient outcomes) trial. In this multicenter, double-blind, randomized trial, ticagrelor (180 mg loading dose, 90 mg twice daily thereafter) significantly reduced the rate of death from vascular causes, myocardial infarction, or stroke without an increase in the rate of overall major bleeding but with an increase in the rate of nonprocedure-related bleeding as compared to clopidogrel (300 to 600 mg loading dose, 75 mg daily thereafter) [87]. 

Cangrelor has been directly compared to clopidogrel in patients with ACS undergoing PCI in two phase II trials (CHAMPION PLATFORM and CHAMPION PCI). Cangrelor was not found to be superior to placebo as far as the composite rate of death, myocardial infarction, or ischemia-driven revascularization at 48 hours but decreased the number of in-stent thromboses at 48 h and death from any cause [88, 89].

Elinogrel was evaluated as an adjunct medication in patients receiving PCI for ACS in the ERASE-MI phase II trial, with no reported increase in adverse effect (bleeding) as compared to placebo [29].

### 5.1. Patients with UA/NSTEMI

The 2012 ACCF/AHA Focused Update of the Guideline for the Management of Patients with Unstable Angina/Non-ST Elevation Myocardial Infarction recommends clopidogrel or ticagrelor in patients with UA/NSTEMI at the time of presentation, regardless of whether PCI is planned or not [83].

For patients undergoing PCI, clopidogrel 600 mg, prasugrel 60 mg, or ticagrelor 180 mg should be administered ideally prior to the procedure. For these patients, clopidogrel 75 mg, prasugrel 10 mg, or ticagrelor 90 mg twice daily are recommended to be continued for 12 months regardless of whether they receive a bare metal or a drug eluting stent [83, 90].

For patients with UA/NSTEMI selected for conservative therapy, either clopidogrel or ticagrelor should be administered for 12 months [83].

The 2011 ESC Guidelines for the management of acute coronary syndromes in patients presenting without persistent ST-segment elevation recommend ticagrelor (180 mg loading dose, 90 mg twice daily) for all patients at moderate-to-high risk of acute non-ST elevation coronary events or prasugrel (60 mg loading dose, 10 mg daily dose) for P2Y_12_-inhibitor-naive patients (especially diabetics) in whom coronary anatomy is known and who are proceeding to PCI, if there is no risk of life-threatening bleeding (class IB). For patients not able to take ticagrelor or prasugrel, the ESC guidelines recommend a 300 mg loading dose of clopidogrel for patients medically treated and 600 mg if PCI is planned [91].

### 5.2. Patients with STEMI

The 2009 ACC/AHA Focused Update on the Guidelines for the Management of Patients with ST Elevation Myocardial Infarction recommends a loading dose of clopidogrel (300–600 mg) or prasugrel (60 mg) in patients with STEMI undergoing PCI [82]. At the time of the guidelines publication, ticagrelor had not been FDA approved. The 2012 ESC guidelines recommend prasugrel and ticagrelor (class IB) or clopidogrel (class IC) as peri-PCI therapeutic adjuncts [92]. 

There is evidence that even in patients presenting with STEMI, treated with fibrinolysis and not with primary PCI, clopidogrel reduces the risk of MACE [93, 94].

Tailoring the antiplatelet therapy to the results of genetic testing or platelet function testing is not routinely recommended. Current guidelines suggest that these tests should be considered only in patients with high risk of poor outcomes. When such patients are identified, changing the therapy from clopidogrel to prasugrel or ticagrelor is a class IIB recommendation [45, 90].

## 6. Conclusions

Dual antiplatelet therapy is the cornerstone long-term treatment for patients presenting with ACS. Premature discontinuation of antiplatelet therapy leads to dire consequences. The significant failure rate of clopidogrel therapy has led to extensive research on the pharmacogenetics of the drug as well as on the role of platelet function testing in adjusting the antiplatelet therapy. Currently, these tests are not routinely recommended. However, in high-risk patients treated with potent P2Y_12_ inhibitors, a personalized antiplatelet therapy tailored according to the results of platelet assays may prove to be beneficial in lowering the risk of spontaneous or perioperative bleeding while not increasing the thrombosis risk. The introduction on the market of newer antiplatelet agents, like ticagrelor or prasugrel, may obviate the need for such testing. If FDA approved, the novel reversible, intravenous P2Y_12_ inhibitors (cangrelor or elinogrel) may become potential therapeutic options especially in the perioperative and periprocedural setting where the current oral agents need to be stopped days in advance in order to prevent excessive bleeding. 

## Figures and Tables

**Table 1:** Studies assessing genetic polymorphism and response to clopidogrel.

Reference	Subjects	Clopidogrel dose	Observed LoF polymorphic alleles	Outcomes
Barker et al. [95]	41 subjects with CAD who had received 75 mg MD for >7 days or <7 days following ≥300 mg LD	LD: ≥300 mg MD: 150 mg	CYP2C19∗2, ∗3, ∗4	Although no statistically significant relation between LoF alleles and high on-treatment platelet reactivity was found, authors observed a tendency toward diminished reduction of this factor in patients with two copies of the LoF allele

Bonello et al. [96]	411 patients with non-ST elevation ACS undergoing PCI	LD: 600 mg, tailored according to the VASP index	CYP2C19∗2	The VASP index significantly higher in carriers of at least one ∗2 allele

Bauer et al. [97]	1024 CLP pretreated patients with CAD undergoing elective coronary stenting	LD: 300 or 600 mg	CYP2C19∗2	Patients with the CYP2C19∗2 allele showed higher on-treatment platelet reactivity

Brandt et al. [59]	89 healthy subjects of predominantly Caucasian origin	LD: 300 mg	CYP2C19∗2 CYP2C9∗2, ∗3 CYP3A4∗1B CYP3A5∗2, ∗3 CYP1A2∗1D, ∗1F, ∗1L CYP2B6∗15A	Significant influence of CYP2C19∗2 and CYP2C9 LoF alleles was observed on poor CLP response (measured as IPA) as well as lower AUC and *C*_max⁡_ of the active metabolite. No association was found between other observed genetic variants and decreased response to CLP

Collet et al. [98]	110 patients from the CLOVIS-2 study	LD: 300 or 900 mg MD: 75 mg	CYP2C19∗2, ∗4	Lowest reduction in platelet aggregation and reduced pharmacokinetic response was associated with the presence of CYP2C19∗2 variant

Collet et al. [99]	259 patient aged <45 years who survived MI of mainly White European or North African origin	MD: ≥75 mg	CYP2C19∗2,∗3, ∗4	CYP2C19∗2 had a strong impact on cardiovascular outcomes expressed as cardiovascular death, MI, or urgent revascularization (adjusted hazard ratio 5.38)

Fontana et al. [100]	94 healthy subjects of Caucasian origin	LD: 300 mg MD: 75 mg	CYP2C19∗2 CYP3A4 (IVS10 + 12A)	Subjects with CYP2C19∗2 had lower reduction in platelet aggregation than wt (*P* = 0.001), whereas no association between pharmacodynamic effect and CYP3A4 (IVS10 + 12A) was observed

Frére et al. [48]	603 patients with non-ST elevation ACS	LD: 600 mg	CYP2C19∗2 CYP3A4∗1B	CYP2C19∗2 allele carriers had higher platelet reactivity to ADP and were more likely to be “nonresponders” (*P* = 0.03). CYP3A4∗1B did not influence either platelet reactivity or platelet response to CLP

Geisler et al. [101]	237 patients of Caucasian origin after implantation of stents owing to symptomatic CAD	LD: 600 mg MD: 75 mg	CYP2C19∗2, ∗3 CYP3A4∗1B CYP3A5∗3	Patients with at least one CYP2C19∗2 had an increased risk of developing high RPA (*P* < 0.0001). No significant association between CYP3A4∗1B and CYP3A5∗3 on RPA

Giusti et al. [102]	1472 patients with acute coronary syndrome	LD: 600 mg MD: 75 mg	CYP2C19∗2 CYP3A4 IVS10 + 12G > A	Only CYP2C19∗2 was associated with higher platelet reactivity after stimulation with ADP and arachidonic acid

Giusti et al. [103]	772 patients enrolled in the RECLOSE trial with acute coronary syndromes	LD: 600 mg MD: 75 mg	CYP2C19∗2	CYP2C19∗2 was an independent factor of stent thrombosis and a composite end point for stent thrombosis and cardiac mortality

Gladding et al. [104]	60 patients undergoing elective PCI enrolled in the PRINC study	LD: 600 or split 1200 mg MD: 75 or 150 mg	CYP2C19∗2, ∗4 CYP2C9∗2, ∗3 CYP3A5∗3	CYP2C19∗2 and ∗4 carriers had attenuated response to CLP. No other observed CYP450 polymorphism had a significant impact on platelet inhibition

Harmsze et al. [105]	428 patients undergoing coronary stent implantation either on chronic CLP maintenance therapy or receiving a LD of CLP	LD: 300 mg MD: 75 mg	CYP2C19∗2 CYP2C9∗2, ∗3 CYP3A4∗1B	Both CYP2C19∗2 and CYP2C9∗3 were associated with attenuated response to CLP and higher platelet reactivity. The modulating effect of CYP2C9∗3 was present only in patients receiving a 300 mg LD of CLP (10-fold increase of risk of poor response). Impact of CYP3A4∗1B genetic variant on response was not observed

Hochholzer et al. [106]	760 patients from EXCELSIOR study	LD: 600 mg MD: 75 mg	CYP2C19∗2	CYP2C19∗2 can be described as a predictor for high on-treatment RPA (*P* < 0.001)

Hulot et al. [107]	28 healthy subjects	MD: 75 mg	CYP2C19∗2, ∗3, ∗4, ∗5, ∗6 CYP3A5∗3 CYP2B6∗5 CYP1A2∗1F	Response to CLP (expressed as ADP-induced platelet aggregation) was strongly influenced by CYP2C19 genotype while other studied polymorphisms had no significant impact

Jinnai et al. [108]	30 patients of Japanese origin scheduled for PCI	LD: 300 mg MD: 75 mg	CYP2C19∗2, ∗3 CYP3A4 (IVS10 + 12G > A)	The IPA values of intermediate and poor metabolizers were significantly lower than that of extensive metabolizers (*P* = 0.04 and *P* = 0.02, resp.). No differences were found for the CYP3A4 allele

Kim et al. [109]	62 patients of CLP arm of ACCELAMI2C19 study of East Asian origin	LD: 600 mg MD: 150 mg	CYP2C19∗2, ∗3	Presence of LoF allele significantly affected platelet reactivity in a 30-day followup after 20 *μ*mol/L ADP-induced LTA but not in 5 *μ*mol/L ADP-induced LTA

Lee et al. [110]	387 patients after PCI of Korean origin undergoing DAT or TAT antiplatelet therapy	LD: 300 mg MD: 75 mg	CYP1A1∗2 CYP1A2 1545T > C CYP2C19∗2, ∗3 CYP3A4 (IVS7 + 268A > G), (IVS10 + 12G > A) CYP3A5∗3 CYP2J2 A > T (rs2046934)	Only CYP2C19∗3 allele was significantly more prevalent in the CLP-resistant group in both dual and triple antiplatelet therapy (*P* = 0.01)

Maeda et al. [111]	97 Japanese patients with CAD in CLP branch of the study	Data not shown	CYP2C19∗2, ∗3	Platelet aggregation significantly higher in poor and intermediate metabolizers

Mega et al. [63]	162 healthy subjects and 1477 patients with ACS from TRITON-TIMI 38 trial with planned PCI	LD: 300 mg MD: 75 mg	CYP2C19∗2A, ∗3, ∗4, ∗5A, ∗6, ∗8, ∗9, ∗10 CYP2C9∗2A, ∗3A, ∗6, ∗9, ∗11A, ∗12 CYP2B6∗6, ∗9, ∗13 CYP3A5∗2A, ∗3A, ∗3F, ∗6 CYP1A2∗1C, ∗1D, ∗1E	Carriers of the reduced-function allele of CYP2C19 and CYP2B6 tended to have lower exposure to the active metabolite of CLP. Difference was also observed in the pharmacodynamic effect expressed as reduction of maximal platelet aggregation. Moreover, CYP2C19 LoF alleles carriers had higher risk for MI, stroke, or death from cardiovascular causes. No association between other CYP450 genotypes and clinical outcomes was found

Oh et al. [112]	2146 patients of East Asian origin treated with PCI	LD: 300 or 600 mg MD: 75 mg	CYP2C19∗2	Carriers of CYP2C19∗2 had a higher on-treatment platelet reactivity (*P* < 0.001). Although presence of LoF allele had no significant impact on revascularization, MI, cardiac death, or MACE separately, composite hard outcome was higher in the carrier group (*P* = 0.009)

Park et al. [113]	114 patients of Korean origin diagnosed with ACS	MD: 75 or 150 mg	CYP2C19∗2, ∗3	Lower plasma concentrations of CLP in CYP2C19 LoF carriers compared to wt genotype (*P* = 0.0574)

Park et al. [114]	236 patients receiving DAT and 238 patients receiving TAT of East Asian origin	LD: 300–600 mg MD: 75 mg	CYP2C19∗2, ∗3	Carriers of LoF allele in DAT group had lower platelet inhibition as compared to the noncarriers but not in the TAT group

Pettersen et al. [115]	219 patients from the ASCET substudy with stable CAD	MD: 75 mg	CYP2C19∗2	Higher prevalence of CLP resistance was observed in CYP2C19∗2 carriers

Simon et al. [116]	2208 patient from the FAST-MI study	LD: 300 mg (mean) MD: 75 mg (mean)	CYP2C19∗2, ∗3, ∗4, ∗5 CYP3A5∗3	LoF alleles of CYP2C19 were associated with increased risk of death, MI, or stroke

Shuldiner et al. [117]	429 healthy Amish persons and 227 patients undergoing PCI	Amish: LD: 300 mg; MD: 75 mg for 6 days Patients: LD: 300 or 600 mg MD: 75 mg	Genome-wide study including SNP with allele frequency greater than 1%, including CYP2C19 ∗2, ∗3, ∗5	Higher platelet aggregation in CYP2C19∗2 carriers after therapy with clopidogrel (both Amish and patient groups) and higher cardiovascular event rate after 1-year followup in the patient group

Umemura et al. [118]	47 healthy subjects of Japanese origin	LD: 300 mg	CYP2C19∗2, ∗3	Intermediate and poor metabolizers had lower response to CLP and lower values of AUC and *C*_max⁡_ of CLP active metabolite than extensive metabolizers

Wallentin et al. [51]	5148 patients in the CLP arm of the PLATO trial	LD: 300–600 mg MD: 75 mg	CYP2C19∗2, ∗3, ∗4, ∗5, ∗6, ∗8	Higher rates of cardiovascular events at 30 days in carriers of LoF allele

Angiolillo et al. [119]	82 patients of Caucasian origin with stable CAD and 45 CLP-naive patients	LD: 300 mg MD: 75 mg	CYP3A4∗1B, IVS7 + 258A > G, IVS7 + 894C > T, IVS10 + 12G > A	IVS10 + 12G > A had an influence on platelet activation; however, it did not have effect on the platelet aggregation profile

Suh et al. [120]	32 healthy volunteers and 348 patients after coronary angioplasty with stent implantation of Korean origin	LD: 300 mg MD: 75 mg	CYP3A5∗3	In the healthy group, CYP3A5∗3 did not alter significantly platelet inhibition. In the patients group, atherothrombotic events occurred more frequently in carriers of CYP3A5∗3

Smith et al. [121]	Total 94 patients listed for elective PCI	LD: 300 or 600 mg MD: 75 mg	CYP3A5∗3	Studied variation did not significantly influence CLP antiplatelet response

ACS: acute coronary syndrome; ADP: adenosine diphosphate; AUC: area under the curve concentration versus time; CAD: coronary artery disease; CLP: clopidogrel; DAT: dual antiplatelet therapy; GoF: gene alleles coding an enzyme with gain of function; IPA: inhibition of platelet aggregation; LD: loading dose; LoF: gene alleles coding an enzyme with loss of function; LTA: light transmittance aggregometry; MD: maintenance dose; MI: myocardial infarction; PCI: percutaneous coronary intervention; RPA: residual platelet aggregation; SNP: single nucleotide polymorphism; TAT: triple antiplatelet therapy; VASP: vasodilator-stimulated phosphoprotein (from [42]).

**Table 2:** Characteristics of platelet function assays [122].

Assay	LTA	MEA	VerifyNow P2Y_12_	VASP	Impact-R	PFA 100 INNOVANCE	Platelet works
Measurement	Aggregation	Aggregation	Aggregation	Activation	Adhesion/aggregation	Aggregation	Aggregation
Specimen type	Platelet-rich plasma	Whole blood	Whole blood	Whole blood	Whole blood	Whole blood	Whole blood
Standard conditions	Variable ADP concentration	Variable ADP concentration	Fixed ADP and PGE1	Fixed ADP and PGE1	Recommended ADP	Fixed PGE1	Recommended ADP
Technical skills required	Specialized lab and personnel	Specialized lab and personnel	None	Specialized lab and personnel	Specialized lab and personnel	None	None
Advantages	Well-studied tied to clinical outcomes	Physiologic environment Minimal sample manipulation	User-friendly and fast No sample processing	P2Y_12_ receptor-specific Small blood volume required	User-friendly and fast Small blood volume required	User-friendly and fast Small blood volume required	Fast
Disadvantages	Time and extent of processing lack of standardization	Less clinical evidence with assay smaller range for response	Unadjustable agonist concentration expensive	Measures earlier marker (signaling)	Limited experience with assay	Result dependent on von Willebrand factor levels and hematocrit	Time dependent Not well studied

**Table 3:** Outcome studies with different inhibition of platelet aggregation.

Study	Study patients	Clopidogrel dose	Platelet function assay	Platelet reactivity measure	End-point prediction
Matetzky et al. [123]	STEMI-PCI patients: 60	300 mg post-PCI	LTA (ADP-induced aggregation)	Patients stratified into 4 quartiles	MACE at 6 months *P* < 0.01
Gurbel et al. [124]	ELECTIVE PCI, patients: 192	300/600 mg post PCI	LTA and TEG	Patients stratified in different quartiles	MACE at 6 months *P* = 0.02
Bliden et al. [125]	ELECTIVE PCI, patients: 100	75 mg for >1 month	LTA and TEG	Preprocedural platelet aggregation in patients on clopidogrel	MACE at 12 months *P* < 0.001
Bonello et al. [96]	ELECTIVE PCI, patients: 144	300 mg, 24 hours prior to PCI	VASP-P	PRI > 50%	MACE at 6 months *P* < 0.01
Price et al. [73]	ELECTIVE PCI, patients: 380	600 mg, 12 hours prior to PCI	VerifyNow	PRU > 235	MACE at 6 months *P* = 0.008
Marcucci et al. [74]	ACS-PCI patients: 683	600 mg prior to PCI	VerifyNow	PRU > 240	MACE at 12 months CV death *P* = 0.034 MI *P* = 0.004
Migliorini et al. [126]	PCI-unprotected LM, patients: 215	600 mg prior to PCI	LTA	Platelet reactivity > 70%	MACE at 19.3 months *P* = 0.005
El Ghannudi et al. [127]	ELECTIVE and URGENT PCI, patients: 461	300 or 600 mg	VASP-P	PRI > 61%	MACE at 9 months *P* = 0.037
Breet et al. [79]	ELECTIVE PCI, patients: 1069	75 mg >5 days or 300 mg >24 hours prior or 600 mg >4 hours prior to PCI	LTA, VerifyNow, Plateletworks, IMPACT, Innovance PFA and PFA-100	Standard platelet function measurement values	MACE at 12 months

ADP: adenosine diphosphate; LM: left main; LTA: light transmittance aggregometry; CV: cardiovascular; MACE: major adverse cardiovascular events; MI: myocardial infarction; PFA: platelet function assay; PRI: platelet reactivity index; PRU: P2Y_12_ reaction unit; TEG: thrombelastography; VASP-P: vasodilator-stimulated phosphoprotein phosphorylation. From [75].
